# Comparison of appendicular lean mass indices for predicting physical performance in Korean hemodialysis patients: A cross-sectional study

**DOI:** 10.1097/MD.0000000000028168

**Published:** 2021-12-10

**Authors:** Jun Chul Kim, Jun Young Do, Ji-Hyung Cho, Seok Hui Kang

**Affiliations:** a Division of Nephrology, Department of Internal Medicine, CHA Gumi Medical Center, CHA University, Gumi, Republic of Korea; b Division of Nephrology, Department of Internal Medicine, Yeungnam University Medical Center, Daegu, Republic of Korea.

**Keywords:** hemodialysis, lean mass index, physical performance, strength

## Abstract

Few studies have examined the optimal adjustment indices for predicting low muscle strength or physical performance in hemodialysis (HD) patients. Thus, the present study aimed to identify optimal adjustment indices for predicting strength and/or physical performance in HD patients.

Our study was performed at an HD center (n = 84). Appendicular lean mass (ALM; kg) was calculated using dual-energy X-ray absorptiometry. ALM were adjusted to body weight, height^2^ (Ht^2^), body surface area, or body mass index. Physical performance tests (sit-to-stand test performed 5 times test, sit-to-stand for 30 second test, 6-minute walk test, timed up and go test, gait speed, hand grip strength, average steps per day (AST), and short physical performance battery) were also evaluated. Participants with a below median value for each physical performance test were defined as the low group.

The mean participant age was 55.6 ± 12.8 years; 44 (52.4%) were men. The univariate analysis revealed a significant difference in ALM/Ht^2^ values between the low and normal physical performance group in all physical performance tests except short physical performance battery. The multivariate analysis revealed a significant difference in ALM/Ht^2^ between the low and normal physical performance groups in hand grip strength, 5 times sit-to-stand test, sit-to-stand for 30-second test, and AST. In women on HD, most indices were not associated with physical performance or strength.

We demonstrated that, in men on HD, ALM/Ht^2^ may be the most valuable among various variables adjusted for ALM for predicting physical performance or strength.

## 1 Introduction

Chronic kidney disease is a common public health problem, and chronic kidney disease can progress to end-stage renal disease requiring dialysis or transplantation. Hemodialysis (HD) is the most commonly used treatment modality in patients with end-stage renal disease. Data from the United States Renal Data System and Korean registries showed that 87.9% of end-stage renal disease patients in the United States and 77% in Korea are on HD.^[1,2]^ HD patients are exposed to various pathologic conditions, including uremia, chronic inflammation, and malnutrition.^[3]^ These lead to sarcopenia defined as decreased muscle mass and/or decreased physical performance.^[4–8]^ This is associated with high morbidity and mortality rates among HD patients.^[9–12]^ Therefore, the early prediction and intervention of decreases in muscle mass are essential to improving the prognosis of HD patients.

In HD patients, a definite conclusion regarding optimal muscle mass measurements is lacking, but dual-energy X-ray absorptiometry (DEXA) is the most popular and acceptable method for predicting muscle mass.^[4–8]^ Because raw DEXA data do not consider body size, various adjustment indices using variables such as body weight (BW), body surface area (BSA), or body mass index (BMI) have been used. Previous studies showed an association between various variables such as adjusted index and physical performance or metabolic disturbances in the general population.^[13–17]^ These showed different prevalence values of sarcopenia or abilities to predict physical performance among various variable-adjusted indices. However, differences among nations, ethnicities, and diseases should be considered. Few studies have examined the optimal adjustment indices for predicting low muscle strength or physical performance in HD patients. Thus, the present study aimed to identify optimal adjustment indices for predicting strength and/or physical performance in HD patients.

## 2 Materials and methods

### 2.1 Study population

**Figure 1:**
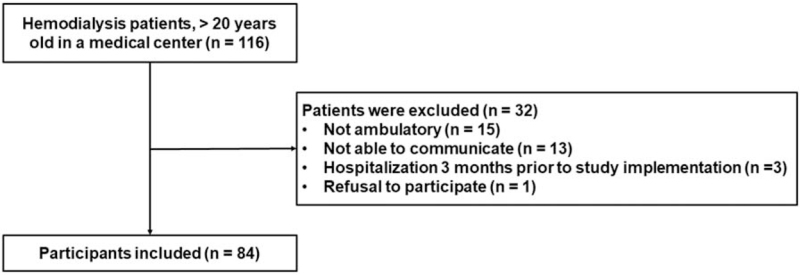
Flowchart of the study.

Our study was performed at an HD center in the Republic of Korea between January and December at 2015 (Fig. 1). We included participants who were >20 years old with a dialysis history ≥6 months, with no history of hospitalization in the previous 3 months, who were able to ambulate without an assistive device, who were able to communicate with the interviewer, and who were able to provide informed consent. Finally, a total of 84 HD patients were included. None of the participants were taking opioids, antihistamines, or antidepressants, which can be associated with decreased physical activity and cognitive function. This study was approved by the Institutional Review Board of CHA Gumi Medical Center (number: 12–07) that follows the principles of the Declaration of Helsinki. All participants provided written informed consent.

### 2.2 Study variables

Laboratory or demographic data collected at enrollment included sex, age, hemoglobin level (g/dL), Kt/*V*_urea_, high-sensitivity C-reactive protein (mg/dL), serum albumin level (g/dL), dialysis vintage, and the presence of diabetes mellitus (DM). Kt/*V*_urea_ was calculated using Daugirdas formula.^[18]^ If a patient had a self-reported history and a medical record of a DM diagnosis or medication use, the patient was defined as having DM.

Muscle mass measurements were performed using DEXA. Measurements were performed after the midweek HD session. DEXA was measured using a Prodigy Advance instrument and performed by a technologist while each participant was in a supine position and clothed in a light gown (GE Medical Systems Lunar, Madison, WI). Appendicular lean mass (ALM; kg) was calculated using the sum of both upper and lower extremities. ALM were adjusted to BW (kg), height^2^ (Ht^2^; m^2^), BSA (m^2^), or BMI (kg/m^2^). BSA was calculated using the DuBois formula.^[19]^ BMI was calculated as BW divided by Ht^2^.

The fat mass (FM) index (kg/m^2^) was calculated using the total FM determined from the DEXA measurements and height squared. In addition, computed tomography (Aquilion ONE; Toshiba Medical Systems Corp., Tokyo, Japan) images of each participant were obtained at the mid-thigh level, specifically at the midpoint of a line extending from the superior border of the patella to the greater trochanter (3-mm thickness, 5 slices). The thigh muscle area (TMA, cm^2^; –190 to –30 Hounsfield unit [HU]) and thigh intermuscular fat area (IMFA, cm^2^; –29 to 100 HU) were measured from the images using ImageJ software (version 1.5e; National Institutes of Health, Bethesda, MD). Thigh muscle quality (%) was calculated using 100 × IMFA/(IMFA + TMA). Sarcopenia was defined as both low muscle mass and low strength (for ALM/Ht^2^, <7.0 kg/m^2^ in men and <5.4 kg/m^2^ in women; for hand grip strength [HGS], <28 kg in men and <18 kg in women).^[7]^

### 2.3 Assessment of physical performance

In our study, physical performance data included the 5-times sit-to-stand test (5STS), sit-to-stand for 30-second test (STS30), 6-minute walk test (6MWT), timed up and go test (TUG), gait speed (GS), and HGS. These tests were performed using standard protocols.^[20–23]^ Briefly, each participant started the 5STS test in a seated position in a chair with the arms crossed and hands touching the shoulders. The participants were asked to stand up and sit down 5 times as quickly as possible, and the time taken to do so in seconds was recorded. The STS 30 test was started with each participant in a seated position in a chair with their arms crossed and hands touching the shoulders. Values were defined as the number of stands a person could complete in 30 seconds without using their arms to stand. The 6MWT was scored as follows: participants were asked to walk at their best pace for 6 minutes, and the distance they covered was recorded in meters. The TUG test was recorded as follows: the participants were instructed to rise from an arm-chair, walk 3 m, turn around, return, and sit down, and the time was recorded in seconds. The HGS test was performed using a manual hydraulic dynamometer (Jamar hydraulic hand dynamometer; Sammons Preston, Chicago, IL) and recorded for maximum strength during the 3 trials on the dominant hand. The average steps per day (AST) were calculated using a pedometer.

The short physical performance battery (SPPB) test was calculated using previously defined methods from the GS, 5STS, and balance test (scored between 0 and 12).^[24]^ We also defined a low physical performance group for each physical performance test. Participants with a below median value for each physical performance test were defined as the low group.

### 2.4 Statistical analyses

SPSS version 21.0 was used to analyze the data (SPSS, Chicago, IL, USA). Categorical variables are expressed as both counts and percentages. Continuous variables are expressed as mean ± standard deviation or standard error. For continuous variables, means were compared using Student *t* test. A multivariate analysis was performed using analysis of covariance. Correlations between 2 continuous variables were assessed using Pearson or partial correlation analyses. Linear regression analysis was performed to assess the independent predictors of each physical performance. The multivariate analysis was adjusted for age and DM. The level of statistical significance was set at *P* < .05.

## 3 Results

### 3.1 Participants’ clinical characteristics

The mean participant age was 55.6 ± 12.8 years (Table 1); 44 (52.4%) were men. No significant differences were noted in the variables between the male and female participants. The mean ALM, ALM/BW, ALM/Ht^2^, ALM/BSA, and ALM/BMI values were 19.9 ± 3.0 kg, 30.4 ± 4.0%, 7.12 ± 0.82 kg/m^2^, 11.4 ± 1.2 kg/m^2^, and 0.85 ± 0.13 kg/kg/m^2^ in men, and 14.6 ± 2.3 kg, 25.6 ± 3.6%, 5.99 ± 0.77 kg/m^2^, 9.3 ± 1.0 kg/m^2^, and 0.63 ± 0.11 kg/kg/m^2^ in women, respectively (men vs women; *P* < .001 for all variables). The mean 5STS, STS30, 6MWT, TUG, AST, GS, HGS, and SPPB values were 9.2 ± 8, 18.4 ± 6.1, 473 ± 121, 7.2 ± 2.2, 4660 ± 3160, 0.97 ± 0.22, 29.9 ± 7.2, and 10.9 ± 1.7 in men and 8.5 ± 2.6, 17.2 ± 5.2, 443 ± 103, 7.5 ± 1.8, 5178 ± 3762, 0.86 ± 0.16, 21.8 ± 4.8, and 10.9 ± 1.5 in women (men vs women; *P* = .630, *P* = .326, *P* = .230, *P* = .577, *P* = .500, *P* = .013, *P* < .001, and *P* = .975, respectively). In the male and female participants, the BMIs were 23.7 ± 3.4 and 23.7 ± 3.9 kg/m^2^ (*P* = .970), the FM indices were 5.7 ± 2.7 and 7.8 ± 2.9 kg/m^2^ (*P* = .001), the TMAs were 109.6 ± 21.6 and 83.6 ± 16.4 cm^2^ (*P* < .001), the IMFAs were 5.1 ± 3.7 and 6.0 ± 5.8 cm^2^ (*P* = .355), and the thigh muscle qualities were 4.4 ± 3.0% and 6.5 ± 5.4% (*P* = .030), respectively. The number of patients with sarcopenia was 13 (15.5%), 10 (22.7%) of whom were men and 3 (7.5%) were women (*P* = .072).

**Table 1:** Participants’ clinical characteristics.

	Total (n = 84)	Male (n = 44)	Female (n = 40)	*P*-value
Age, yr	55.6 ± 12.8	55.8 ± 11.4	57.2 ± 12.6	.593
Sex (male, %)	44 (52.4%)	–	–	
Dialysis vintage, yr	4.6 ± 5.1	4.0 ± 4.3	5.2 ± 5.9	.283
Diabetes mellitus (%)	44 (52.4%)	24 (54.5%)	20 (50%)	.677
Hemoglobin, mg/dL	11.0 ± 0.6	11.0 ± 0.7	10.9 ± 0.5	.498
Serum albumin, g/dL	3.8 ± 0.3	3.8 ± 0.3	3.9 ± 0.3	.712
Calcium (mg/dL)	8.4 ± 0.7	8.3 ± 0.7	8.5 ± 0.7	.159
Phosphorus, mg/dL	5.4 ± 1.3	5.4 ± 1.3	5.4 ± 1.3	.897
High sensitivity C-reactive protein, mg/dL	0.4 ± 0.6	0.5 ± 0.7	0.4 ± 0.5	.472
Kt/Vurea	1.36 ± 0.31	1.31 ± 0.37	1.42 ± 0.21	.125

### 3.2 Comparison of differences in indices by physical performance group

The median 5STS, STS30, 6MWT, TUG, AST, GS, HGS, and SPPB values were 7.9, 18.0, 494, 6.5, 4087, 1.01, 30.0, and 12.0 in men and 7.8, 18.0, 466, 6.9, 4292, 0.89, 22.0, and 11.0 in women, respectively. For men, differences in indices are shown Table 2. The univariate analysis revealed a significant difference in ALM/Ht^2^ values between the low and normal physical performance group in all physical performance tests except SPPB. The multivariate analysis revealed a significant difference in ALM/Ht^2^ between the low and normal physical performance groups in HGS, 5STS, STS30, and AST. For men, ALM/Ht^2^ was the most discriminating index. For women, most indices had no statistical significance (Supplement 1, Supplemental Digital Content). The associations between muscle mass indices and physical performance were more weakened in multivariate analyses than in univariate analyses. Regarding differences in indices according to groups, some indicators were significant in univariate analysis but not in multivariate analysis (for men, difference were found in the associations between ALM and GS or 6MWT; ALM/BW and HGS; ALM/Ht^2^ and GS, 6MWT, or TUG; and ALM/BSA and GS or 6MWT; and for women, the association between ALM/BMI and 6MWT).

**Table 2:** Difference in appendicular lean mass indices by physical performance group in men.

	ALM		ALM/BW		ALM/Ht^2^		ALM/BSA		ALM/BMI	
	Mean ± SD	*P*-value	Mean ± SD	*P*-value	Mean ± SD	*P*-value	Mean ± SD	*P*-value	Mean ± SD	*P*-value
Univariate										
SPPB										
Low	19.5 ± 3.3	.334	29.6 ± 3.8	.210	6.90 ± 0.78	.086	11.1 ± 1.2	.104	0.83 ± 0.14	.442
Normal	20.4 ± 2.8		31.1 ± 4.2		7.32 ± 0.82		11.7 ± 1.21		0.87 ± 0.13	
GS										
Low	18.9 ± 2.4	.023	29.3 ± 3.7	.090	6.80 ± 0.64	.008	10.9 ± 1.1	.010	0.82 ± 0.13	.104
Normal	21.0 ± 3.3		31.4 ± 4.2		7.44 ± 0.86		11.9 ± 1.2		0.88 ± 0.13	
HGS										
Low	18.2 ± 2.4	<.001	28.9 ± 3.5	.029	6.67 ± 0.82	<.001	10.7 ± 1.1	<.001	0.79 ± 0.11	.007
Normal	21.4 ± 2.9		31.6 ± 4.1		7.49 ± 0.61		12.0 ± 1.0		0.90 ± 0.14	
5STS										
Low	21.1 ± 3.3	.013	31.0 ± 3.9	.314	7.50 ± 0.79	.001	11.9 ± 1.2	.013	0.87 ± 0.13	.376
Normal	18.8 ± 2.3		29.7 ± 4.2		6.74 ± 0.66		11.0 ± 1.1		0.83 ± 0.14	
STS30										
Low	19.0 ± 2.9	.061	30.0 ± 4.5	.602	6.82 ± 0.81	.020	11.1 ± 1.4	.088	0.84 ± 0.16	.614
Normal	20.8 ± 3.0		30.7 ± 3.6		7.39 ± 0.74		11.7 ± 1.0		0.86 ± 0.11	
6MWT										
Low	19.0 ± 2.6	.044	29.6 ± 3.9	.220	6.81 ± 0.72	.011	11.0 ± 1.2	.027	0.83 ± 0.13	.280
Normal	20.9 ± 3.3		31.1 ± 4.1		7.43 ± 0.81		11.8 ± 1.2		0.87 ± 0.13	
TUG										
Low	20.8 ± 3.4	.076	30.8 ± 4.0	.451	7.44 ± 0.82	.011	11.8 ± 1.2	.060	0.86 ± 0.13	.631
Normal	19.1 ± 2.6		29.9 ± 4.1		6.83 ± 0.71		11.1 ± 1.2		0.84 ± 0.14	
AST										
Low	19.2 ± 3.1	.118	29.3 ± 3.6	.105	6.76 ± 0.75	.004	11.0 ± 1.2	.015	0.83 ± 0.12	.364
Normal	20.6 ± 2.9		31.3 ± 4.2		7.45 ± 0.74		11.8 ± 1.1		0.87 ± 0.15	
Multivariate										
SPPB										
Low	19.9 ± 0.6	.985	30.0 ± 0.9	.643	7.00 ± 0.17	.387	11.3 ± 0.3	.539	0.85 ± 0.03	.804
Normal	19.9 ± 0.6		30.6 ± 0.8		7.22 ± 0.17		11.5 ± 0.2		0.84 ± 0.03	
GS										
Low	19.3 ± 0.6	.170	29.6 ± 0.8	.229	6.88 ± 0.16	.055	11.1 ± 0.2	.069	0.83 ± 0.03	.400
Normal	20.5 ± 0.6		31.1 ± 0.8		7.35 ± 0.16		11.7 ± 0.2		0.87 ± 0.03	
HGS										
Low	18.4 ± 0.5	<.001	29.1 ± 0.8	.055	6.71 ± 0.15	.001	10.8 ± 0.2	<.001	0.80 ± 0.03	.014
Normal	21.2 ± 0.5		31.4 ± 0.8		7.46 ± 0.14		11.9 ± 0.2		0.89 ± 0.02	
5STS										
Low	20.8 ± 0.6	.048	30.8 ± 0.8	.439	7.45 ± 0.15	.004	11.8 ± 0.2	.033	0.86 ± 0.03	.677
Normal	19.1 ± 0.6		29.9 ± 0.8		6.79 ± 0.15		11.1 ± 0.2		0.84 ± 0.03	
STS30										
Low	19.2 ± 0.6	.087	30.1 ± 0.8	.624	6.85 ± 0.16	.025	11.1 ± 0.2	.100	0.84 ± 0.03	.733
Normal	20.6 ± 0.6		30.6 ± 0.8		7.36 ± 0.15		11.7 ± 0.2		0.86 ± 0.03	
6MWT										
Low	19.6 ± 0.6	.489	30.0 ± 0.9	.597	6.92 ± 0.17	.121	11.2 ± 0.3	.259	0.85 ± 0.03	.949
Normal	20.3 ± 0.6		30.7 ± 0.9		7.32 ± 0.17		11.6 ± 0.3		0.85 ± 0.03	
TUG										
Low	20.2 ± 0.6	.576	30.3 ± 0.9	.979	7.33 ± 0.17	.110	11.6 ± 0.3	.433	0.83 ± 0.03	.472
Normal	19.7 ± 0.6		30.4 ± 0.9		6.92 ± 0.17		11.3 ± 0.2		0.86 ± 0.03	
AST										
Low	19.3 ± 0.6	.179	29.5 ± 0.8	.145	6.79 ± 0.15	.006	11.0 ± 0.02	.023	0.84 ± 0.03	.521
Normal	20.5 ± 0.6		31.2 ± 0.8		7.42 ± 0.15		11.8 ± 0.02		0.86 ± 0.03	

### 3.3 Association between various indices and physical performance tests

In men, correlation coefficients are shown Table 3. Pearson correlation coefficients for ALM/Ht^2^ and ALM/BSA were statistically significant in all physical performances except 5STS for ALM/Ht^2^ and STS30 for ALM/BSA. Partial correlation coefficients for ALM/Ht^2^ were statistically significant in GS, 6MWT, TUG, and AST. Those for ALM/BSA were statistically significant in GS, 6MWT, and AST. In women, most indices showed no statistical significance in the correlation analyses (Supplement 2, Supplemental Digital Content). Linear regression analyses showed similar trends with results from the correlation analyses (Table 4 and Supplement 3, Supplemental Digital Content). In correlation or linear regression analyses, some indicators were significant in univariate analysis but not in multivariate analysis (for men, significant differences were found in the associations between ALM and GS, HGS, 6MWT, TUG, or AST; ALM/BW and SPPB or GS; ALM/Ht^2^ and SPPB, HGS, or STS30; and ALM/BSA and SPPB, HGS, 5STS, or TUG; and for women, they were between ALM/BW or ALM/BMI and TUG).

**Table 3:** Correlation between appendicular lean mass indices and physical performance in men.

	ALM		ALM/BW		ALM/Ht^2^		ALM/BSA		ALM/BMI	
	*r*	*P*-value	*r*	*P*-value	*r*	*P*-value	*r*	*P*-value	*r*	*P*-value
Pearson correlation										
SPPB	0.221	.149	0.315	.037	0.347	.021	0.369	.014	0.235	.125
GS	0.407	.006	0.325	.031	0.473	.001	0.470	.001	0.310	.041
HGS	0.368	.014	0.203	.187	0.374	.012	0.367	.014	0.259	.089
5STS	−0.215	.161	−0.273	.073	−0.296	.051	−0.322	.033	−0.219	.153
STS30	0.191	.215	0.144	.352	0.301	.047	0.250	.101	0.084	.590
6MWT	0.436	.003	0.419	.005	0.524	<.001	0.552	<.001	0.387	.009
TUG	−0.365	.015	−0.189	.218	−0.453	.002	−0.383	.010	−0.180	.243
AST	0.311	.043	0.432	.004	0.422	.005	0.487	.001	0.372	.014
Partial correlation										
SPPB	0.098	.543	0.191	.231	0.242	.128	0.237	.136	0.084	.603
GS	0.296	.060	0.266	.093	0.393	.011	0.392	.011	0.200	.211
HGS	0.226	.155	0.040	.806	0.233	.143	0.192	.230	0.073	.648
5STS	−0.081	.614	−0.180	.261	−0.187	.241	−0.200	.210	−0.087	.587
STS30	0.022	.890	0.007	.967	0.171	.286	0.082	.612	−0.107	.505
6MWT	0.245	.123	0.334	.033	0.395	.011	0.422	.006	0.211	.165
TUG	−0.222	.164	−0.059	.712	−0.341	.029	−0.238	.135	0.000	1.000
AST	0.302	.055	0.424	.006	0.417	.007	0.496	.001	0.359	.021

**Table 4:** Linear regression analyses of physical performances by appendicular lean mass indices in men.

	ALM		ALM/BW		ALM/Ht^2^		ALM/BSA		ALM/BMI	
	Ust-β (SE)	*P*	Ust-β (SE)	*P*	Ust-β (SE)	*P*	Ust-β (SE)	*P*	Ust-β (SE)	*P*
Univariate										
SPPB	0.13 (0.09)	.149	0.14 (0.06)	.037	0.74 (0.31)	.021	0.53 (0.20)	.014	3.05 (1.95)	.125
GS	0.03 (0.01)	.006	0.02 (0.01)	.031	0.12 (0.04)	.001	0.08 (0.02)	.001	0.50 (0.24)	.041
HGS	0.86 (0.34)	.014	0.36 (0.27)	.187	3.28 (1.26)	.012	2.15 (0.84)	.014	13.85 (7.96)	.089
5STS	−0.57 (0.40)	.161	−0.55 (0.30)	.073	−2.96 (1.47)	.051	−2.14 (0.97)	.033	−13.33 (9.156)	.153
STS30	0.38 (0.30)	.215	0.22 (0.23)	.352	2.26 (1.10)	.047	1.25 (0.75)	.101	3.81 (7.02)	.590
6MWT	17.2 (5.5)	.003	12. 6 (4.2)	.005	77.6 (19.5)	<.001	54.5 (12.7)	<.001	348.5 (128.1)	.009
TUG	−0.27 (0.10)	.015	−0.11 (0.08)	.218	−1.24 (0.38)	.002	−0.70 (0.26)	.010	−2.98 (2.52)	.243
AST	319 (153)	.043	336 (109)	.004	1621 (543)	.005	1247 (350)	.001	8705 (3395)	.014
Multivariate										
SPPB	0.08 (0.09)	.432	0.09 (0.07)	.191	0.56 (0.33)	.099	0.38 (0.23)	.102	1.43 (2.08)	.494
GS	0.02 (0.01)	.113	0.01 (0.01)	.125	0.09 (0.04)	.021	0.06 (0.03)	.026	0.25 (0.24)	.307
HGS	0.60 (0.37)	.110	0.10 (0.27)	.709	2.19 (1.33)	.107	1.28 (0.91)	.169	5.40 (8.32)	.520
5STS	−0.33 (0.46)	.471	−0.41 (0.32)	.210	−2.20 (1.62)	.183	−1.62 (1.10)	.151	−7.47 (10.04)	.461
STS30	0.16 (0.34)	.641	0.06 (0.25)	.814	1.59 (1.21)	.195	0.67 (0.83)	.424	−2.56 (7.49)	.734
6MWT	10.3 (5.9)	.089	9.70 (4.42)	.025	57.2 (20.2)	.007	41.6 (13.6)	.004	206.5 (131.2)	.123
TUG	−0.17 (0.12)	.146	−0.04 (0.09)	.668	−0.94 (0.40)	.025	−0.45 (0.29)	.120	−0.25 (2.62)	.925
AST	362 (183)	.055	356 (122)	.006	1771 (619)	.007	1460 (409)	.001	9394 (3914)	.021

In men, the correlation coefficients between the FM index and TMA or IMFA were 0.204 and 0.710, respectively (*P* = .184 for TMA and *P* < .001 for IMFA), and in women, these were 0.468 and 0.507, respectively (*P* = .002 for TMA and *P* = .001 for IMFA).

## 4 Discussion

On uni- and multivariate analyses, our study showed consistent superiority of ALM/Ht^2^ for predicting physical performance or strength compared with the other indices in men on HD. In women on HD, most indices were not associated with physical performance or strength. The relationship between 2 continuous variables can be evaluated using correlation and/or linear regression analyses. Correlation analysis can simply show the directional nature and strength of the relationship between 2 variables. Linear regression analysis is useful in identifying the extent of influence for dependent variables caused by independent variables. In the male participants, partial correlation analysis showed that ALM/Ht^2^ was positively correlated with GS, 6MWT, and AST but was inversely correlated with TUG. These revealed that the increase in ALM/Ht^2^ is consistently associated with better results in some physical performances, including GS, 6MWT, TUG, and AST. Furthermore, multiple linear regression analyses show that 1-unit increases of ALM/Ht^2^ are numerically associated with an increase in 0.09 m/s of GS, increase in 57.2 m of 6MWT, decrease in 0.94 seconds of TUG, and increase in 1771 steps of AST.

Janssen et al^[4]^ showed a positive association between the BW-adjusted muscle mass index and physical function. The European Working Group, International Working Group, and Asian Working Group recommended the use of a height-adjusted muscle mass index,^[5–7]^ whereas the Foundation Institutes of Health Biomarker Consortium most recently recommended the use of a BMI-adjusted muscle mass index.^[8]^ Many consensuses recommended use of a height-adjusted muscle mass index. Kittiskulnam et al^[25]^ compared sarcopenia and physical performance according to various variable-adjusted muscle mass indices in HD patients. They showed that a height-adjusted index underestimates sarcopenia, especially in patients with a high BMI, and emphasized the limitation of a height-adjusted index. However, BW-adjusted indies are prone to the effect of fat mass. Previous studies showed that BW-adjusted muscle mass would be favorably associated with disability and/or cardiometabolic disturbances and height-adjusted muscle mass would be favorably associated with physical performance.^[4,26–28]^

Previous studies of Korean populations investigated an association between various muscle mass indices and cardiovascular disease or metabolic disturbances.^[29–31]^ Lim et al^[29]^ enrolled elderly Koreans and compared a BW- or height-adjusted muscle mass index with metabolic syndrome and showed superiority of the former. Park et al^[30]^ enrolled Korean adults and showed a positive association between a BW-adjusted index and stroke risk in men. Byeon et al^[31]^ showed a positive association between a BW-adjusted index and cardiovascular risk score in Korean adults. These studies enrolled elderly or middle-aged healthy populations. Therefore, studies using disease-, nation-, and ethnicity-specific populations are needed to determine the optimal adjusted index for predicting physical performance. Our study enrolled Korean HD patients. To our knowledge, the present study was the first to evaluate the association between various variable-adjusted lean mass indices and physical performance or strength in Korean HD patients.

Our data showed significant differences in ALM indices, GS, and HGS between men and women. We performed subgroup analyses by sex and showed that ALM/Ht^2^ had the best predictability for physical performance and strength in men. A height-adjusted index was one of the most popular adjustment for body size. This adjustment can underestimate real muscle mass in patients with a high BMI; however, in our study, mean BMI in men and women was 23.7 ± 3.4 and 23.7 ± 3.9 kg/m^2^, respectively, within the normal range.^[32]^ The relative normal BMI value of our cohort would lead to a positive association between a height-adjusted index and physical performance or strength. However, no indices were associated with the physical performance and strength in the female participants, possibly due to the decreased muscle quality.

In our study, there were no significant associations between muscle mass indices and strength or physical performances in the female participants compared with those in the male participants. Kim et al^[33]^ reported a positive association between muscle mass indices and physical performance status in only weak populations. Muscle mass is positively associated and intramuscular or intermuscular fat is inversely associated with strength or physical performance.^[34–36]^ Muscle dysfunction, such as strength and performance, can be determined earlier compared with decreased in muscle mass; this may be associated with the increased inter- and intra-muscular fat infiltration.^[37–39]^ In our cohort, the female participants had less muscle mass and greater muscular fat compared with men. In addition, the FM index was positively associated with both TMA and IMFA in women. Both fat and protein, such as muscle mass, are more important nutritional indicators in HD patients than in the general population, and muscle mass can increase along with an increase in fat mass.^[40]^ Moreover, in our cohort, the mean age of the female participants was 57.2 ± 12.6 years. Although postmenopausal status was not available in our study, significant proportions of women may be postmenopausal, making them more prone to insulin resistance. In these conditions, women can easily show an increase in fat mass and muscle mass, and our data revealed a positive correlation between the FM index with TMA and IMFA in the female participants alone. Furthermore, the prevalence of sarcopenia in the female participants was very low (7.5%) compared with the 22.7% in the male participants. Consequently, 3 factors—the combination with small muscle mass and high muscular fat, correlation between muscle mass and fat mass, and low prevalence of sarcopenia—may be associated with the lack of a relationship between muscle mass indices and strength or physical performances in women.

HD patients have high insulin resistance compared with the general population, and women have less muscle mass than men.^[37]^ Relatively small muscle mass and high insulin resistance combined with inter- and intra-muscular fat accumulation may be associated with the lack of a relationship between 2 variables in women.

Kittiskulnam et al^[12]^ compared mortality rates among muscle mass indices in HD patients in the USA and showed that all known muscle mass indices at the time were not better in association with mortality compared with physical performance, GS, and strength. The mean BMI in their study was 27.4 kg/m^2^ in men and 29.2 kg/m^2^ in women (28.1 kg/m^2^ in the total cohort). Another study using the same cohort showed that the height-adjusted muscle mass index underestimates the prevalence of low muscle mass among obese patients and was poorer in predicting GS compared with the BW, BSA, and BMI-adjusted muscle mass indices.^[25]^ However, a Japanese study with HD patients with a mean BMI of 21.9 kg/m^2^ showed that height-adjusted muscle mass is associated with all-cause mortality.^[41]^ The BMI values in our cohort were closer to the values reported in the Japanese study than to the values reported in the American studies. These results revealed that BW or BMI-adjusted muscle mass index can be useful in predicting outcomes in HD patients with high BMIs or who are obese. The height-adjusted muscle mass index may be more useful in predicting outcomes in HD patients with normal or low BMIs. To the best of our knowledge, although there are no definite recommendations to date regarding the optimal adjusted muscle mass index to predict outcomes in HD patients, its use may be considered with the BW or BMI-adjusted index in cohorts with high BMIs and the height-adjusted index in cohorts with low or normal BMIs.

Our study has some limitations. First, it was performed in a single center and included a small number of patients. Second, we enrolled all participants who agreed to provide informed consent and did not calculate a proper sample size for statistical significance. Third, the low groups as a categorical variable for SPPB, GS, HGS, 5STS, STS30, 6MWT, TUG, AST were defined using the median value of each variable in our study. There were cut-off values for some indicators, such as SPPB, GS, HGS, 5STS, STS30, or TUG, as previous studies described.^[7,42–46]^ However, HD patients in our cohort were relatively stable, and categories using these values revealed that most of the patients were in the normal group. These resulted in a biased distribution of dependent variables according to the category of independent variables, which was associated with a decrease in the goodness of the regression model. Therefore, we defined the cut-off using the median value, which would be helpful in decreasing the biased distribution of dependent variables. Large sample sized studies that include patients with various conditions would be needed to secure the goodness of the regression model despite a biased distribution. Analyses using broad ranges of muscle mass, physical performance, and strength would be more informative. Fourth, analyses without the adjustment of additional confounding factors were inherent limitations of our study. We did not perform multivariate analyses using sufficient confounding factors. Most variables, such as hemoglobin, serum albumin, serum calcium, inflammation, or dialysis adequacy, may be associated with muscle mass or physical activities via direct or indirect pathways. Our study had a small sample size, and adjustment using many variables may lead to statistical non-significance. However, patients in our study were relatively stable without a wide variation of these variables, which may be helpful in attenuating these limitations.

Our study had some strengths despite these limitations. It was performed using comprehensive evaluations, including body composition measurements (such as DEXA and computed tomography), strength, and physical performance tests (including SPPB, GS, 5STS, STS30, TUG, and AST). Although the association between these muscle mass indices and each indicator was already evaluated in previous studies, there are few studies to date on the association between muscle mass indices and outcomes, including these comprehensive measurements. In addition, our cohort included HD patients as a specific population, and we identified the differences in associations in terms of sex. These differences in associations in terms of sex between muscle mass indices and strength or physical performances have not yet been fully elucidated in HD patients. It is difficult to form a definite conclusion due to the small sample size, single center, and cross-sectional natures of our study. However, our study may be helpful in recognizing the necessity of further studies regarding the association between muscle mass indices and strength or physical performance in HD patients. A future large prospective study is warranted to overcome these limitations.

## 5 Conclusion

Here we demonstrated that, in men on HD, ALM/Ht^2^ may be the most valuable among various variables adjusted for ALM for predicting physical performance or strength.

## Author contributions

Seok Hui Kang analyzed and interpreted the patient data; and writing the manuscript. Jun Young Do and Ji-Hyung Cho performed generation and collection of data. Seok Hui Kang and Jun Chul Kim performed drafting and revision of the manuscript. All authors read and approved the final manuscript

**Conceptualization:** Seok Hui Kang.

**Data curation:** Jun Chul Kim, Jun Young Do, Ji-Hyung Cho, Seok Hui Kang.

**Formal analysis:** Jun Chul Kim, Seok Hui Kang.

**Funding acquisition:** Jun Young Do.

**Investigation:** Seok Hui Kang.

**Resources:** Ji-Hyung Cho.

**Validation:** Ji-Hyung Cho.

**Writing – original draft:** Seok Hui Kang.

**Writing – review & editing:** Jun Chul Kim.

## Supplementary Material

Supplemental Digital Content

## Supplementary Material

Supplemental Digital Content

## Supplementary Material

Supplemental Digital Content
