# Supplementary material for: Comparison of appendicular lean mass indices for predicting physical performance in Korean hemodialysis patients: A cross-sectional study
Source: Medicine (Baltimore). 2021 Dec 10;100(49):e28168. doi: 10.1097/MD.0000000000028168 (PMC8663833; doi:10.1097/MD.0000000000028168)
Supplement: Supplemental Digital Content [file medi-100-e28168-s001.docx]

**Supplement 1. Difference in muscle mass indices by physical performance group in women**

|  | ALM (kg) | | ALM/BW (%) | | ALM/Ht^2^ (kg/[m]^2^) | | ALM/BSA (kg/m^2^) | | ALM/BMI (kg/[kg/m^2^]) | |
| --- | --- | --- | --- | --- | --- | --- | --- | --- | --- | --- |
|  | Mean ± SD | *P*-value | Mean ± SD | *P*-value | Mean ± SD | *P*-value | Mean ± SD | *P*-value | Mean ± SD | *P*-value |
| **Univariate** |  |  |  |  |  |  |  |  |  |  |
| SPPB |  |  |  |  |  |  |  |  |  |  |
| Low | 15.0 ± 3.0 | 0.473 | 25.3 ± 3.9 | 0.702 | 6.17 ± 0.95 | 0.329 | 9.4 ± 1.2 | 0.710 | 0.61 ± 0.14 | 0.697 |
| Normal | 14.4 ± 1.9 |  | 25.7 ± 3.6 |  | 5.91 ± 0.68 |  | 9.3 ± 0.9 |  | 0.63 ± 0.10 |  |
| GS |  |  |  |  |  |  |  |  |  |  |
| Low | 14.7 ± 2.4 | 0.851 | 26.0 ± 0.8 | 0.439 | 6.09 ± 0.78 | 0.403 | 9.4 ± 1.1 | 0.449 | 0.63 ± 0.13 | 0.862 |
| Normal | 14.5 ± 2.1 |  | 25.1 ± 2.7 |  | 5.88 ± 0.75 |  | 9.2 ± 1.0 |  | 0.62 ± 0.09 |  |
| HGS |  |  |  |  |  |  |  |  |  |  |
| Low | 14.5 ± 2.4 | 0.824 | 25.4 ± 4.0 | 0.821 | 5.96 ± 0.78 | 0.826 | 9.3 ± 1.1 | 0.763 | 0.62 ± 0.13 | 0.834 |
| Normal | 14.7 ± 2.1 |  | 25.7 ± 3.4 |  | 6.01 ± 0.78 |  | 9.4 ± 1.0 |  | 0.63 ± 0.09 |  |
| 5STS |  |  |  |  |  |  |  |  |  |  |
| Low | 14.5 ± 2.1 | 0.800 | 26.2 ± 4.1 | 0.288 | 5.94 ± 0.74 | 0.724 | 9.4 ± 1.1 | 0.696 | 0.64 ± 0.13 | 0.303 |
| Normal | 14.7 ± 2.4 |  | 25.0 ± 3.1 |  | 6.03 ± 0.81 |  | 9.3 ± 0.9 |  | 0.61 ± 0.09 |  |
| STS30 |  |  |  |  |  |  |  |  |  |  |
| Low | 14.5 ± 2.3 | 0.708 | 24.6 ± 2.9 | 0.097 | 5.91 ± 0.78 | 0.540 | 9.1 ± 0.8 | 0.185 | 0.60 ± 0.08 | 0.184 |
| Normal | 14.7 ± 2.3 |  | 26.6 ± 4.1 |  | 6.07 ± 0.77 |  | 9.5 ± 1.2 |  | 0.65 ± 0.13 |  |
| 6MWT |  |  |  |  |  |  |  |  |  |  |
| Low | 14.4 ± 2.5 | 0.587 | 24.7 ± 3.9 | 0.156 | 6.03 ± 0.81 | 0.729 | 9.2 ± 1.0 | 0.383 | 0.59 ± 0.10 | 0.045 |
| Normal | 14.8 ± 2.1 |  | 26.4 ± 3.3 |  | 5.95 ± 0.75 |  | 9.5 ± 1.1 |  | 0.66 ± 0.11 |  |
| TUG |  |  |  |  |  |  |  |  |  |  |
| Low | 14.3 ± 1.8 | 0.422 | 26.4 ± 4.2 | 0.204 | 5.83 ± 0.56 | 0.204 | 9.3 ± 1.0 | 0.946 | 0.65 ± 0.13 | 0.184 |
| Normal | 14.9 ± 2.6 |  | 24.9 ± 3.0 |  | 6.14 ± 0.91 |  | 9.3 ± 1.1 |  | 0.60 ± 0.09 |  |
| AST |  |  |  |  |  |  |  |  |  |  |
| Low | 14.8 ± 2.6 | 0.545 | 25.9 ± 4.4 | 0.625 | 6.02 ± 0.85 | 0.816 | 9.4 ± 1.2 | 0.637 | 0.64 ± 0.13 | 0.465 |
| Normal | 14.4 ± 1.9 |  | 25.3 ± 2.9 |  | 5.96 ± 0.71 |  | 9.3 ± 0.9 |  | 0.61 ± 0.09 |  |
| **Multivariate** |  |  |  |  |  |  |  |  |  |  |
| SPPB |  |  |  |  |  |  |  |  |  |  |
| Low | 14.9 ± 0.7 | 0.561 | 25.4 ± 1.1 | 0.849 | 6.08 ± 0.22 | 0.652 | 9.4 ± 0.3 | 0.840 | 0.63 ± 0.03 | 0.978 |
| Normal | 14.5 ± 0.4 |  | 25.7 ± 0.7 |  | 5.95 ± 0.14 |  | 9.3 ± 0.2 |  | 0.63 ± 0.02 |  |
| GS |  |  |  |  |  |  |  |  |  |  |
| Low | 14.6 ± 0.5 | 0.953 | 26.4 ± 0.8 | 0.230 | 6.02 ± 0.17 | 0.801 | 9.4 ± 0.2 | 0.484 | 0.64 ± 0.03 | 0.440 |
| Normal | 14.6 ± 0.5 |  | 24.8 ± 0.9 |  | 5.96 ± 0.18 |  | 9.2 ± 0.2 |  | 0.61 ± 0.03 |  |
| HGS |  |  |  |  |  |  |  |  |  |  |
| Low | 14.5 ± 0.5 | 0.825 | 25.7 ± 0.9 | 0.934 | 5.91 ± 0.17 | 0.562 | 9.3 ± 0.2 | 0.776 | 0.63 ± 0.03 | 0.760 |
| Normal | 14.7 ± 0.5 |  | 25.6 ± 0.8 |  | 6.05 ± 0.16 |  | 9.4 ± 0.2 |  | 0.62 ± 0.02 |  |
| 5STS |  |  |  |  |  |  |  |  |  |  |
| Low | 14.3 ± 0.5 | 0.397 | 26.0 ± 0.9 | 0.591 | 5.93 ± 0.18 | 0.640 | 9.3 ± 0.2 | 0.994 | 0.63 ± 0.03 | 0.844 |
| Normal | 14.9 ± 0.5 |  | 25.3 ± 0.9 |  | 6.05 ± 0.17 |  | 9.3 ± 0.2 |  | 0.62 ± 0.03 |  |
| STS30 |  |  |  |  |  |  |  |  |  |  |
| Low | 14.7 ± 0.5 | 0.833 | 24.8 ± 0.9 | 0.248 | 5.92 ± 0.17 | 0.566 | 9.2 ± 0.2 | 0.351 | 0.62 ± 0.03 | 0.628 |
| Normal | 14.5 ± 0.5 |  | 26.4 ± 0.9 |  | 6.06 ± 0.17 |  | 9.5 ± 0.2 |  | 0.64 ± 0.03 |  |
| 6MWT |  |  |  |  |  |  |  |  |  |  |
| Low | 14.4 ± 0.5 | 0.574 | 24.8 ± 0.9 | 0.256 | 6.00 ± 0.18 | 0.962 | 9.2 ± 0.2 | 0.354 | 0.59 ± 0.03 | 0.122 |
| Normal | 14.8 ± 0.5 |  | 26.3 ± 0.8 |  | 5.98 ± 0.17 |  | 9.5 ± 0.2 |  | 0.65 ± 0.02 |  |
| TUG |  |  |  |  |  |  |  |  |  |  |
| Low | 13.7 ± 0.5 | 0.031 | 26.0 ± 0.9 | 0.580 | 5.68 ± 0.17 | 0.026 | 9.1 ± 0.2 | 0.243 | 0.63 ± 0.03 | 0.795 |
| Normal | 15.4 ± 0.5 |  | 25.2 ± 0.9 |  | 6.27 ± 0.16 |  | 9.5 ± 0.2 |  | 0.62 ± 0.03 |  |
| AST |  |  |  |  |  |  |  |  |  |  |
| Low | 14.6 ± 0.5 | 0.921 | 25.7 ± 0.9 | 0.855 | 5.92 ± 0.17 | 0.560 | 9.3 ± 0.2 | 0.817 | 0.63 ± 0.03 | 0.666 |
| Normal | 14.6 ± 0.5 |  | 25.5 ± 0.8 |  | 6.05 ± 0.16 |  | 9.4 ± 0.2 |  | 0.62 ± 0.02 |  |

Data are expressed as mean ± standard deviation for univariate analysis and mean ± standard error for multivariate analysis. *P* values were tested using the *t*-test for univariate analysis and analysis of covariance for multivariate analysis. Multivariate analysis was adjusted for age and diabetes mellitus.

Abbreviations: ALM, appendicular lean mass; ALM/BW, appendicular lean mass per body weight; ALM/Ht^2^, appendicular lean mass per height squared; ALM/BSA, appendicular lean mass per body surface area; ALM/BMI, appendicular lean mass per body mass index; SPPB, short physical performance battery; Low, low group; Normal, normal group; GS, gait speed; HGS, hand grip strength; 5STS, 5 times sit-to-stand test; STS30, sit-to-stand for 30 seconds test; 6MWT, 6-minute walk test; TUG, timed up and go test; AST, average steps per day.
