# Supplementary material for: Comparison of appendicular lean mass indices for predicting physical performance in Korean hemodialysis patients: A cross-sectional study
Source: Medicine (Baltimore). 2021 Dec 10;100(49):e28168. doi: 10.1097/MD.0000000000028168 (PMC8663833; doi:10.1097/MD.0000000000028168)
Supplement: Supplemental Digital Content [file medi-100-e28168-s002.docx]

**Supplement 2. Correlation between appendicular lean mass indices and physical performance in women**

|  | ALM | | ALM/BW | | ALM/Ht^2^ | | ALM/BSA | | ALM/BMI | |
| --- | --- | --- | --- | --- | --- | --- | --- | --- | --- | --- |
|  | *r* | *P*-value | *r* | *P*-value | *r* | *P*-value | *r* | *P*-value | *r* | *P*-value |
| **Pearson correlation** |  |  |  |  |  |  |  |  |  |  |
| SPPB | –0.089 | 0.586 | 0.104 | 0.523 | -0.140 | 0.389 | –0.017 | 0.919 | 0.119 | 0.464 |
| GS | 0.016 | 0.921 | –0.026 | 0.875 | –0.094 | 0.564 | –0.030 | 0.854 | 0.072 | 0.657 |
| HGS | 0.181 | 0.263 | 0.105 | 0.519 | 0.079 | 0.626 | 0.148 | 0.361 | 0.194 | 0.231 |
| 5STS | –0.048 | 0.769 | –0.204 | 0.207 | –0.061 | 0.709 | –0.156 | 0.336 | –0.182 | 0.262 |
| STS30 | 0.058 | 0.724 | 0.129 | 0.429 | 0.098 | 0.547 | 0.135 | 0.407 | 0.100 | 0.539 |
| 6MWT | –0.082 | 0.613 | 0.235 | 0.145 | –0.224 | 0.164 | 0.027 | 0.867 | 0.2914 | 0.068 |
| TUG | 0.137 | 0.399 | –0.337 | 0.033 | 0.226 | 0.161 | –0.062 | 0.702 | –0.335 | 0.035 |
| AST | 0.023 | 0.889 | 0.008 | 0.962 | 0.129 | 0.435 | 0.073 | 0.658 | –0.051 | 0.760 |
| **Partial correlation** |  |  |  |  |  |  |  |  |  |  |
| SPPB | –0.075 | 0.659 | 0.070 | 0.679 | –0.061 | 0.719 | 0.008 | 0.961 | 0.048 | 0.776 |
| GS | 0.048 | 0.777 | –0.066 | 0.700 | –0.026 | 0.878 | –0.013 | 0.940 | 0.014 | 0.933 |
| HGS | 0.236 | 0.159 | 0.075 | 0.658 | 0.176 | 0.298 | 0.191 | 0.258 | 0.146 | 0.389 |
| 5STS | 0.088 | 0.605 | –0.102 | 0.549 | –0.012 | 0.943 | –0.048 | 0.777 | –0.020 | 0.904 |
| STS30 | –0.055 | 0.745 | 0.022 | 0.896 | 0.074 | 0.664 | 0.038 | 0.823 | –0.063 | 0.711 |
| 6MWT | –0.111 | 0.511 | 0.209 | 0.214 | –0.208 | 0.218 | 0.026 | 0.877 | 0.233 | 0.164 |
| TUG | 0.270 | 0.106 | –0.315 | 0.057 | 0.280 | 0.093 | –0.009 | 0.958 | –0.247 | 0.140 |
| AST | 0.050 | 0.768 | 0.014 | 0.934 | 0.177 | 0.295 | 0.105 | 0.535 | –0.051 | 0.766 |

Partial correlations were adjusted for age and diabetes mellitus.

Abbreviations: ALM, appendicular lean mass; ALM/BW, appendicular lean mass per body weight; ALM/Ht^2^, appendicular lean mass per height squared; ALM/BSA, appendicular lean mass per body surface area; ALM/BMI, appendicular lean mass per body mass index; *r*, correlation coefficient; SPPB, short physical performance battery; Low, low group; Normal, normal group; GS, gait speed; HGS, hand grip strength; 5STS, 5 times sit-to-stand test; STS30, sit-to-stand for 30 seconds test; 6MWT, 6-minute walk test; TUG, timed up and go test; AST, average steps per day.
