# Supplementary material for: Comparison of appendicular lean mass indices for predicting physical performance in Korean hemodialysis patients: A cross-sectional study
Source: Medicine (Baltimore). 2021 Dec 10;100(49):e28168. doi: 10.1097/MD.0000000000028168 (PMC8663833; doi:10.1097/MD.0000000000028168)
Supplement: Supplemental Digital Content [file medi-100-e28168-s003.docx]

**Supplement 3. Linear regression analyses of physical performances by appendicular lean mass indices in women**

|  | ASM | | ASM/BW | | ASM/Ht^2^ | | ALM/BSA | | ALM/BMI | |
| --- | --- | --- | --- | --- | --- | --- | --- | --- | --- | --- |
|  | Ust-β (SE) | *P* | Ust-β (SE) | *P* | Ust-β (SE) | *P* | Ust-β (SE) | *P* | Ust-β (SE) | *P* |
| **Univariate** |  |  |  |  |  |  |  |  |  |  |
| SPPB | –0.06 (0.11) | 0.586 | 0.04 (0.07) | 0.523 | –0.27 (0.32) | 0.389 | –0.02 (0.24) | 0.919 | 1.64 (2.22) | 0.464 |
| GS | 0.00 (0.01) | 0.921 | –0.00 (0.001) | 0.875 | –0.02 (0.03) | 0.564 | –0.01 (0.03) | 0.854 | 0.11 (0.23) | 0.657 |
| HGS | 0.39 (0.34) | 0.263 | 0.14 (0.21) | 0.519 | 0.50 (1.01) | 0.626 | 0.69 (0.75) | 0.361 | 8.52 (6.99) | 0.231 |
| 5STS | –0.06 (0.19) | 0.769 | –0.15 (0.11) | 0.207 | –0.21 (0.55) | 0.709 | –0.40 (0.41) | 0.336 | –4.33 (3.80) | 0.262 |
| STS30 | 0.13 (0.38) | 0.724 | 0.18 (0.23) | 0.429 | 0.67 (1.10) | 0.547 | 0.68 (0.82) | 0.407 | 4.78 (7.72) | 0.539 |
| 6MWT | –3.8 (7.4) | 0.613 | 6.6 (4.4) | 0.145 | –30.0 (21.2) | 0.164 | 2.7 (16.2) | 0.867 | 274.5 (146.1) | 0.068 |
| TUG | 0.11 (0.13) | 0.399 | –0.17 (0.08) | 0.033 | 0.53 (0.37) | 0.161 | –0.11 (0.28) | 0.702 | –5.51 (2.52) | 0.035 |
| AS | 38 (271) | 0.889 | 8 (167) | 0.962 | 624 (792) | 0.435 | 264 (593) | 0.658 | –1722 (5587) | 0.760 |
| **Multivariate** |  |  |  |  |  |  |  |  |  |  |
| SPPB | –0.05 (0.11) | 0.653 | 0.03 (0.06) | 0.667 | –0.12 (0.33) | 0.711 | 0.01 (0.24) | 0.960 | 0.65 (2.17) | 0.765 |
| GS | 0.00 (0.01) | 0.774 | –0.00 (0.001) | 0.694 | –0.01 (0.04) | 0.877 | –0.00 (0.03) | 0.938 | 0.02 (0.24) | 0.935 |
| HGS | 0.51 (0.35) | 0.159 | 0.08 (0.21) | 0.689 | 1.13 (1.06) | 0.291 | 0.89 (0.78) | 0.263 | 5.83 (7.09) | 0.416 |
| 5STS | 0.10 (0.18) | 0.599 | –0.07 (0.11) | 0.540 | –0.04 (0.55) | 0.945 | –0.12 (0.40) | 0.773 | –0.47 (3.63) | 0.898 |
| STS30 | –0.13 (0.38) | 0.740 | 0.03 (0.22) | 0.889 | 0.49 (1.12) | 0.663 | 0.19 (0.83) | 0.820 | –2.75 (7.44) | 0.713 |
| 6MWT | –4.8 (7.2) | 0.511 | 5.1 (4.1) | 0.219 | –26.3 (21.0) | 0.218 | 2.5 (15.9) | 0.877 | 195.6 (139.1) | 0.168 |
| TUG | 0.19 (0.11) | 0.105 | –0.12 (0.06) | 0.059 | 0.57 (0.33) | 0.094 | –0.01 (0.26) | 0.960 | –3.32 (2.23) | 0.145 |
| AS | 93 (311) | 0.768 | 15 (179) | 0.934 | 961 (905) | 0.295 | 423 (676) | 0.535 | –1836 (6113) | 0.766 |

Data are expressed as unstandardized β (standard errors). The multivariate analyses were adjusted for age and diabetes mellitus.

Abbreviations: ASM, appendicular muscle mass; ASM/BW, appendicular muscle mass per body weight; ASM/Ht^2^, appendicular muscle mass per height squared; ALM, appendicular lean mass; ALM/BW, appendicular lean mass per body weight; ALM/Ht^2^, appendicular lean mass per height squared; ALM/BSA, appendicular lean mass per body surface area; ALM/BMI, appendicular lean mass per body mass index; Ust-β, unstandardized beta; SE, standard error; *P*, *P*-value; SPPB, short physical performance battery; GS, gait speed; HGS, hand grip strength; 5STS, 5 times sit-to-stand test; STS30, sit-to-stand for 30 seconds test; 6MWT, 6-minute walk test; TUG, timed up and go test; AST, average steps per day.
